# BactEXTRACT: an R Shiny app to quickly extract, plot and analyse bacterial growth and gene expression data

**DOI:** 10.1099/acmi.0.000742.v3

**Published:** 2024-01-25

**Authors:** Julien Dénéréaz, Jan-Willem Veening

**Affiliations:** ^1^​ Department of Fundamental Microbiology, Faculty of Biology and Medicine, University of Lausanne, Lausanne, CH-1015, Switzerland

**Keywords:** bacterial growth, high-throughput data analysis, microtitre plate reader, RShiny

## Abstract

To streamline the analysis and visualization of bacterial growth and gene expression data obtained by microtitre plate readers, we developed BactEXTRACT, an intuitive, easy-to-use R Shiny application. BactEXTRACT simplifies the transition from raw optical density, fluorescence and luminescence measurements to publication-ready plots. This package offers a user-friendly interface that reduces the complexity involved in growth curve and gene expression analysis and is generally applicable. BactEXTRACT is available at https://veeninglab.com/bactextract.

## Data availability

BactEXTRACT source code along with the most recent development version can be found at https://github.com/veeninglab/BactEXTRACT, and the online R Shiny application is available with any web browser at https://veeninglab.com/bactextract. Example data used in this article can be found at https://github.com/veeninglab/BactEXTRACT/tree/main/Examples.

## Introduction

Bacterial growth measurement is one of the most commonly used methods to assess bacterial fitness and is essential for a broad spectrum of applications, ranging from basic biological research to the screening and development of antibiotics [1]. Bacterial growth is typically assessed by measuring the optical density of a culture over time at a specific wavelength through periodic sampling using a spectrophotometer or within a microplate reader [2]. The advantage of using plate readers is that many strains and conditions can be tested simultaneously. However, such high-throughput experiments yield large datasets that require extensive analysis. As a result, several tools have been developed that can dive deeply into quantifying the growth parameters and can perform detailed statistical analysis [2–7]. In order to simplify the process between output data and producing publish-ready plots, we developed BactEXTRACT, an R Shiny application, to expedite the analysis and visualization of bacterial growth data (https://veeninglab.com/bactextract). This application stands out for its simplicity and intuitive design, aimed at easing the process of growth curve analysis and plotting.

## Functionality

**Fig. 1.**
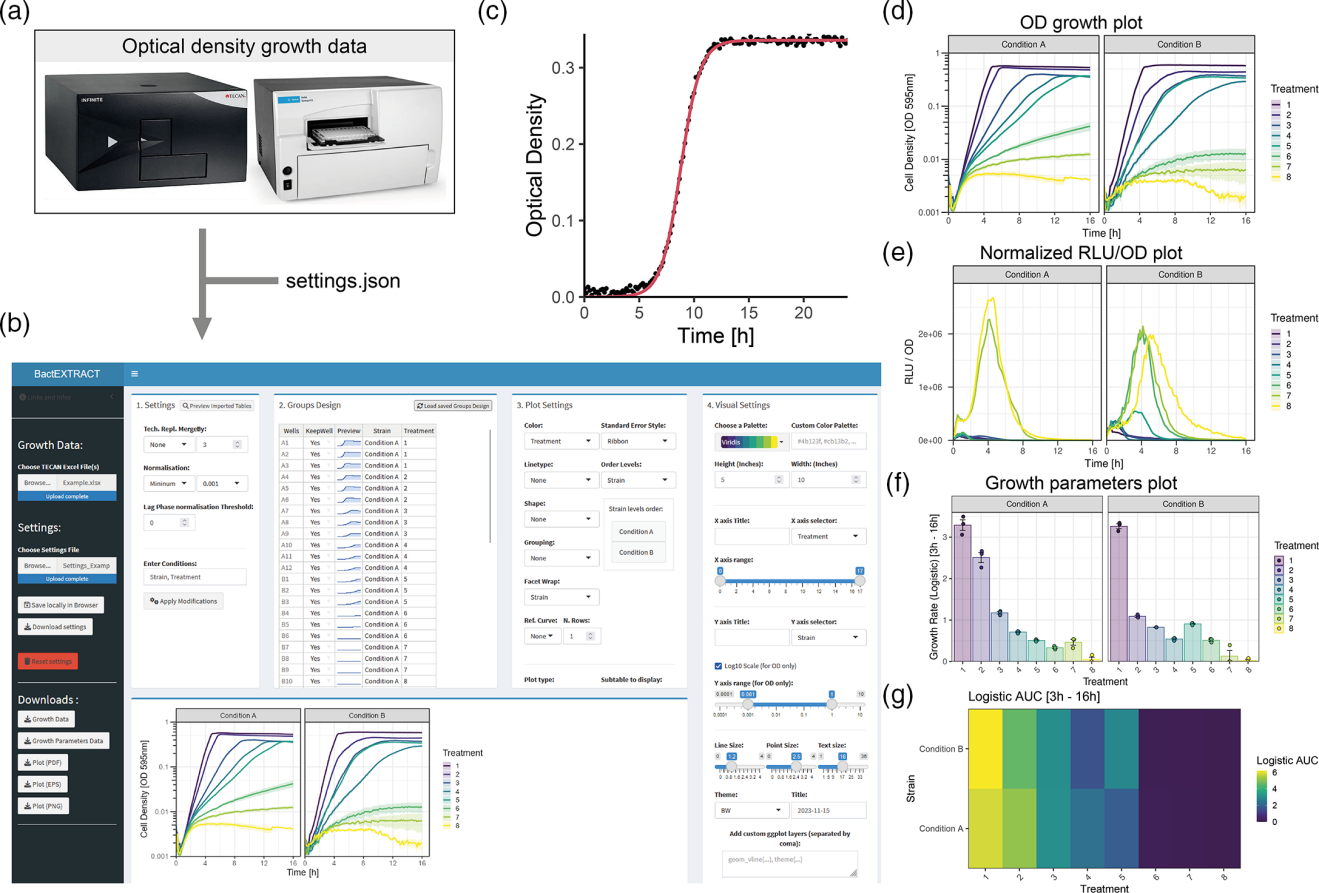
BactEXTRACT R Shiny application. (a) Raw data from common plate readers and simple text files can be input into the software. Multiple files can be uploaded at the same time. (**b)** The web interface of BactEXTRACT allows for the quick editing of any parameters and customized theming. (**c)** BactEXTRACT fits a logistic curve to every sample. Different graphical layers such as colour, shape, linetype and facet can be chosen to allow for clear separation of all groups present in the data. (**d)** Standard OD growth plots on logarithmic or linear axes can be plotted. (**e)** Luminescence and fluorescence sub-tables (if any) can be selected and plotted as well, either normalized by OD or as a standalone plot. (**f, g)** Each growth parameter output from the logistic fitting can be plotted as either a bar plot (**f**) or as a checkerboard plot, which is particularly handy when combinations of antimicrobial compounds are being tested (**g**).

BactEXTRACT is a versatile software tool designed to facilitate the analysis of biological data, particularly optical density (OD) and other related measurements, such as luminescence and fluorescence. It allows for the import of one or multiple Excel or text files, with any amount of sub-table combinations (OD, luminescence, fluorescence), enhancing efficiency in handling extensive datasets. Excel files must be the raw data file produced by common microplate reader such as TECAN or BioTek (Fig. 1a). Currently, supported softwares are TECAN i-control, SparkControl and BioTek Gen5. A settings file can also be uploaded, enabling users to save their analysis parameters, making it possible to consistently apply the same analysis criteria to different datasets without reconfiguring settings each time. Local web browser storage can also be used to save settings that will automatically be set the next time on the same web browser. Customized conditions can easily be entered in the app by the user, allowing the separation of the growth data, for example by strain and treatment (Fig. 1b). In addition, BactEXTRACT allows merging of technical replicates by means of adjacent wells (such as wells A1–A2–A3), allowing, for example, the merging of multiple data files corresponding to biological replicates. The application provides useful normalization settings, either by time or by OD. Normalization by OD enables the use of specific wells as background to use for normalization, and time normalization allows the standardization of lag phases over a dataset. Customization options are abundant and are based on the conditions set by the user (Fig. 1b), affording users the ability to tailor the thematic presentation of their data. It also supports the creation of combination plots that display OD with relative light units (RLU) or with any other fluorescence/luminescence data present in the Excel file normalized by OD, a crucial requirement for reporter growth assays (Fig. 1e). To analyse growth parameters, BactEXTRACT relies on Growthcurver [2], a simple R package fitting a logistic equation to each growth curve using:

,$$N_{t}=\frac{K}{1+\left(\frac{K-N_{0}}{N_{0}}\right)e^{-rt}}$$

where *N_t_* describes the population size at time *t*, as described in the Growthcurver package (Fig. 1c) [2]. The user has the flexibility to select the desired time range for the fitting process. Standard parameters are available for plotting, such as area under the curve (AUC), growth rate and all single parameters of the fitted logistic equation. Visual representations of these parameters include either a bar plot or a checkerboard plot, which is particularly beneficial for contrasting two different conditions, such as in antibiotic combination assays (Fig. 1f, g). Finally, BactEXTRACT allows for the export of both visual plots in various file formats and computed data, facilitating easy sharing and further examination of the results.

## Discussion

BactEXTRACT offers several features that can facilitate scientific research and does not require the acquisition of programming skills that can be time consuming. While BactEXTRACT is very powerful at plotting data, it has a few limitations. For example, currently statistical analysis between groups has to be performed by the user. However, as the data can be downloaded in the ‘tidy’ format, further processing in R is facilitated. Despite offering fewer complexities in mathematical modelling than other bacterial growth curve analysis softwares, such as AMiGA and QurvE [7, 8], BactEXTRACT distinguishes itself with its simplicity and straightforward approach and has become one of the favourite tools used in our group and by our collaborators for analysing and plotting growth curves and we thus expect it to be of general use to the microbiology community. Future developments will allow more features to be added, such as different parametric models to fit bacterial growth curves, or quality of life improvements for better customization plots and faster processing.
